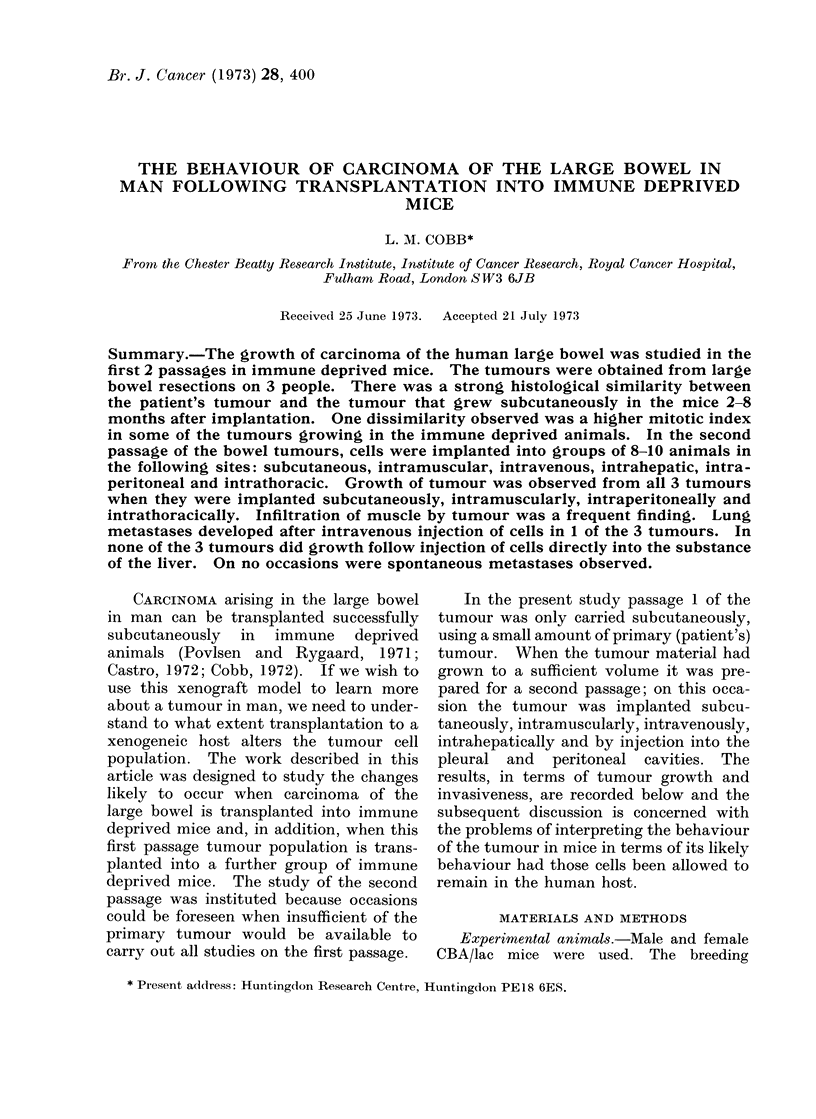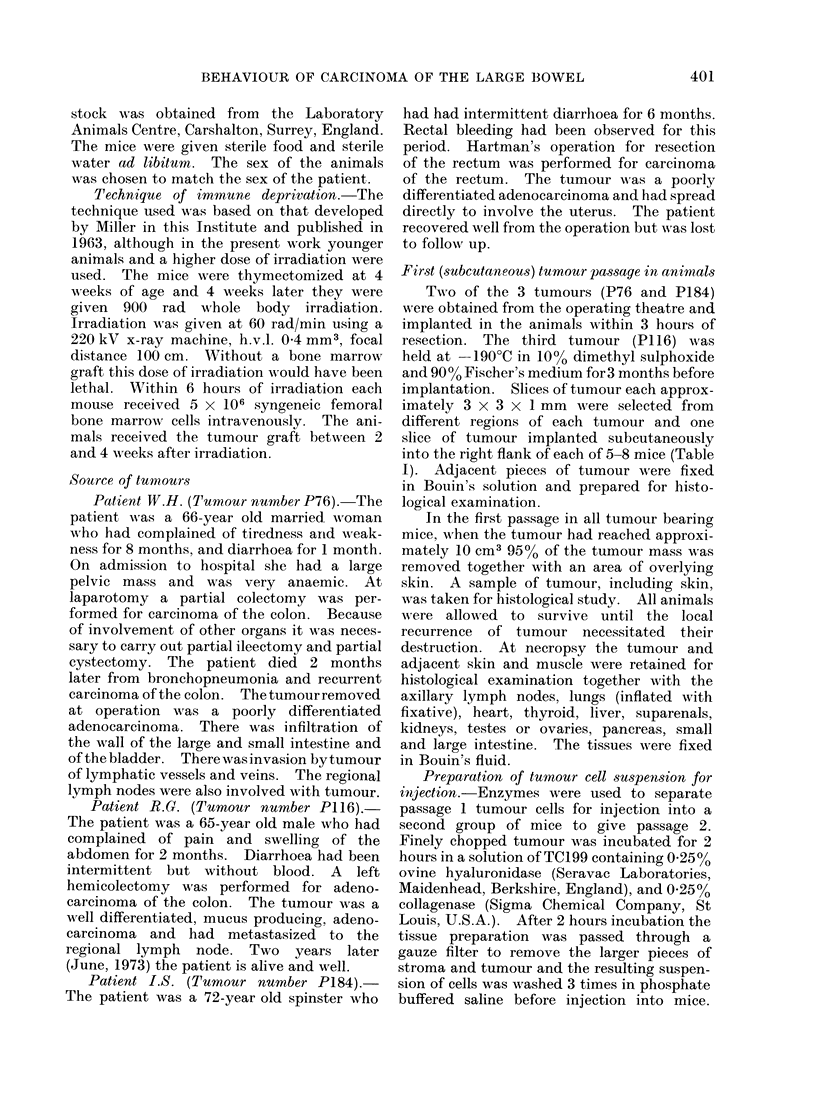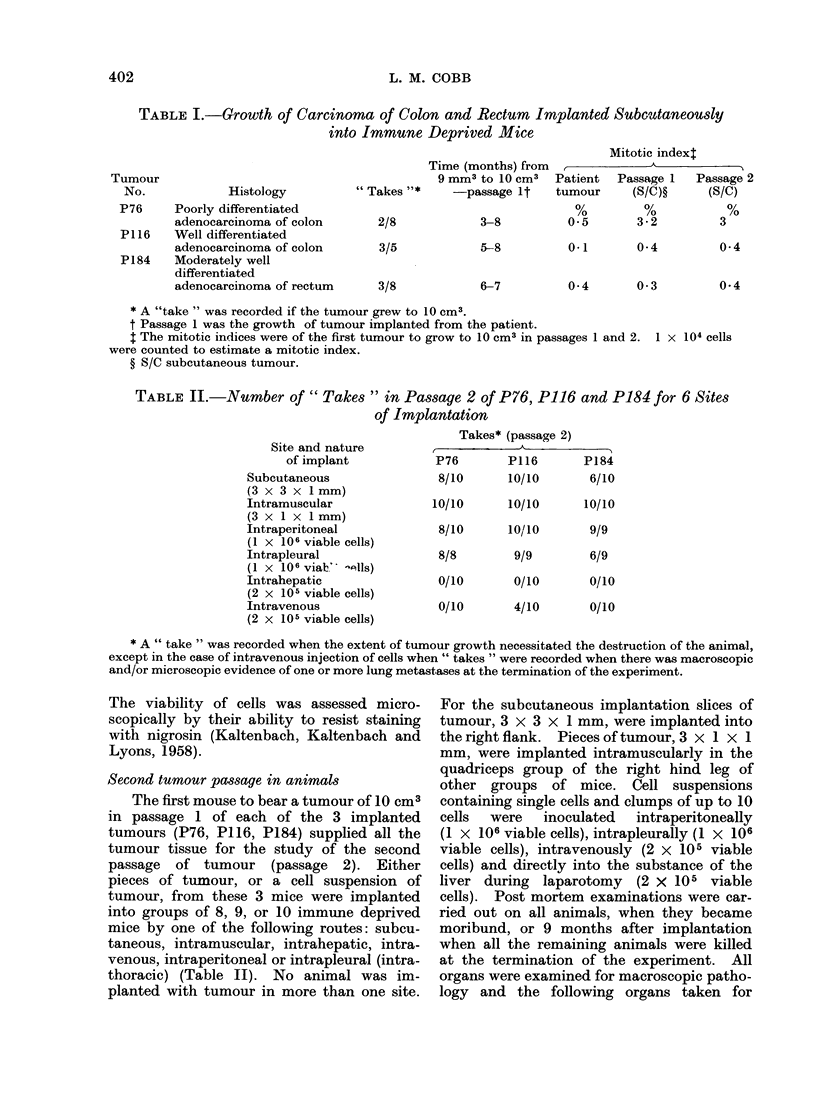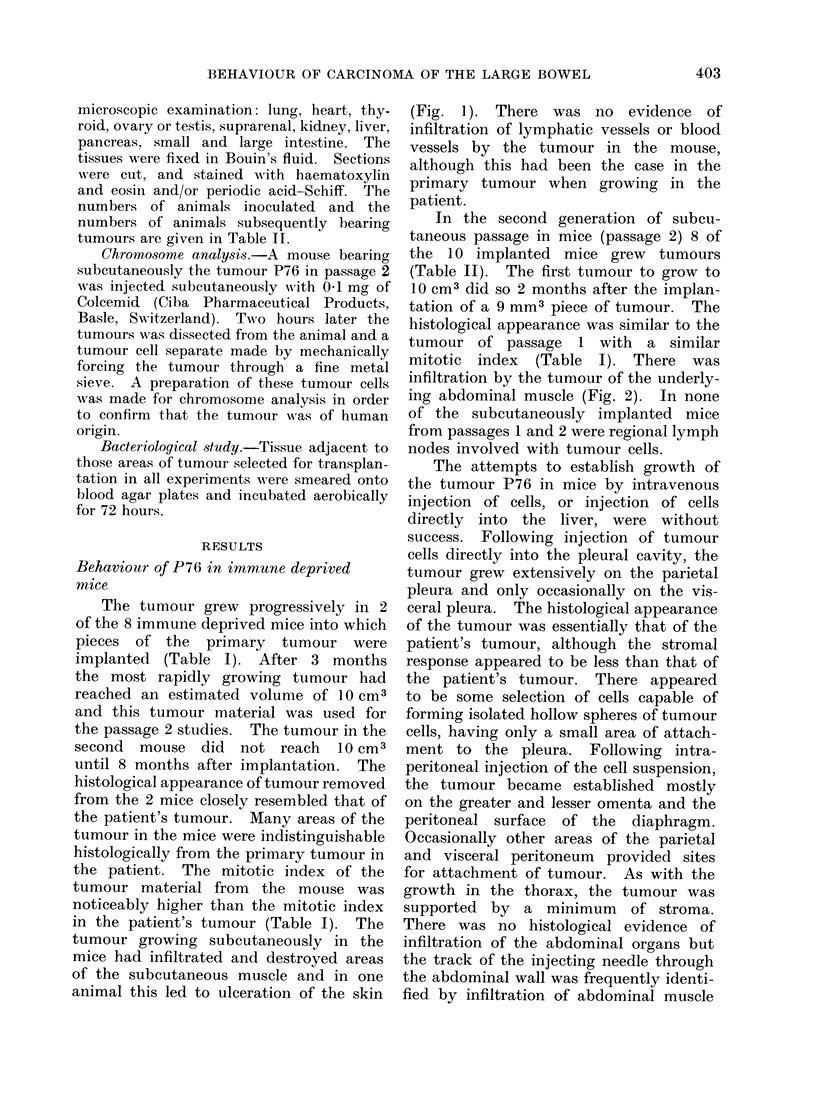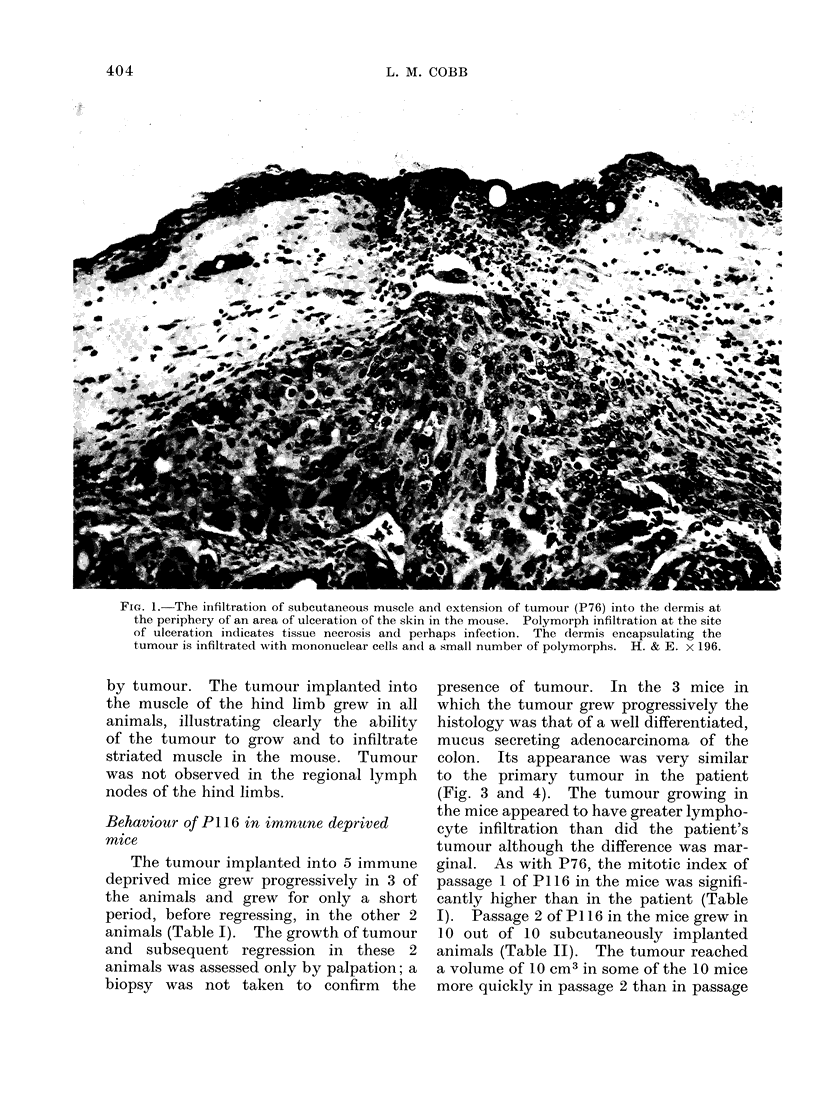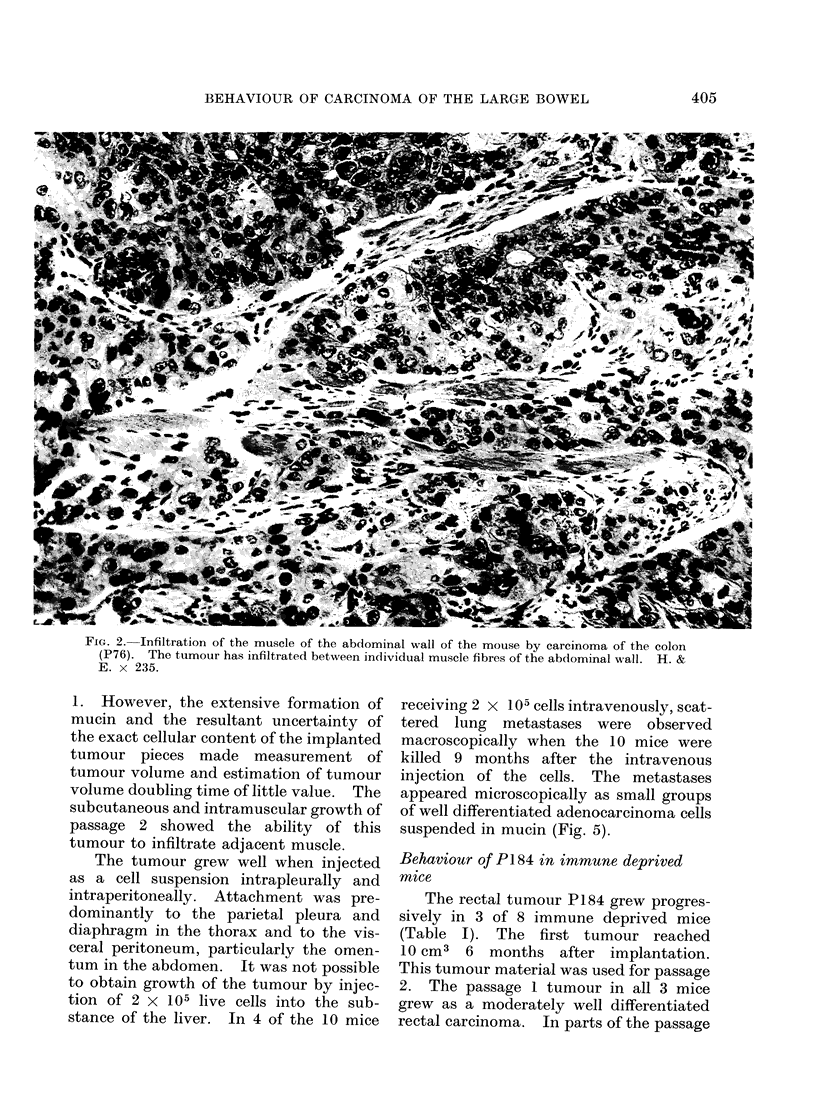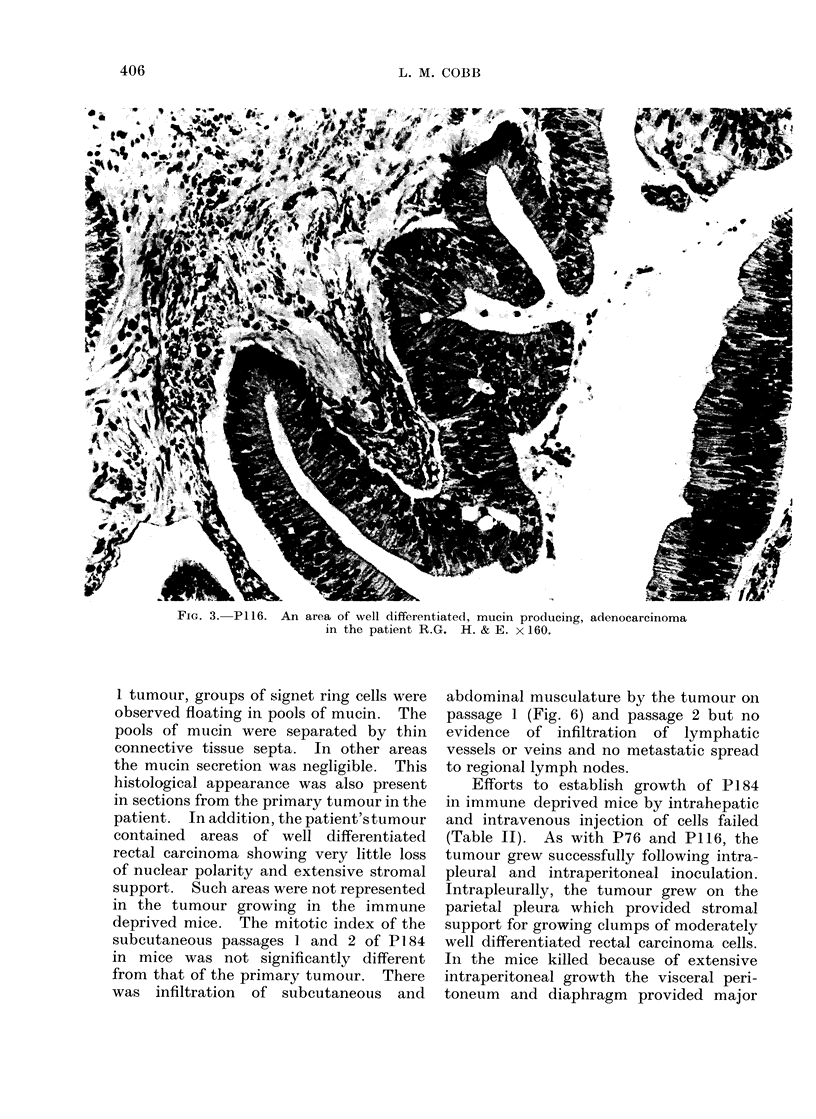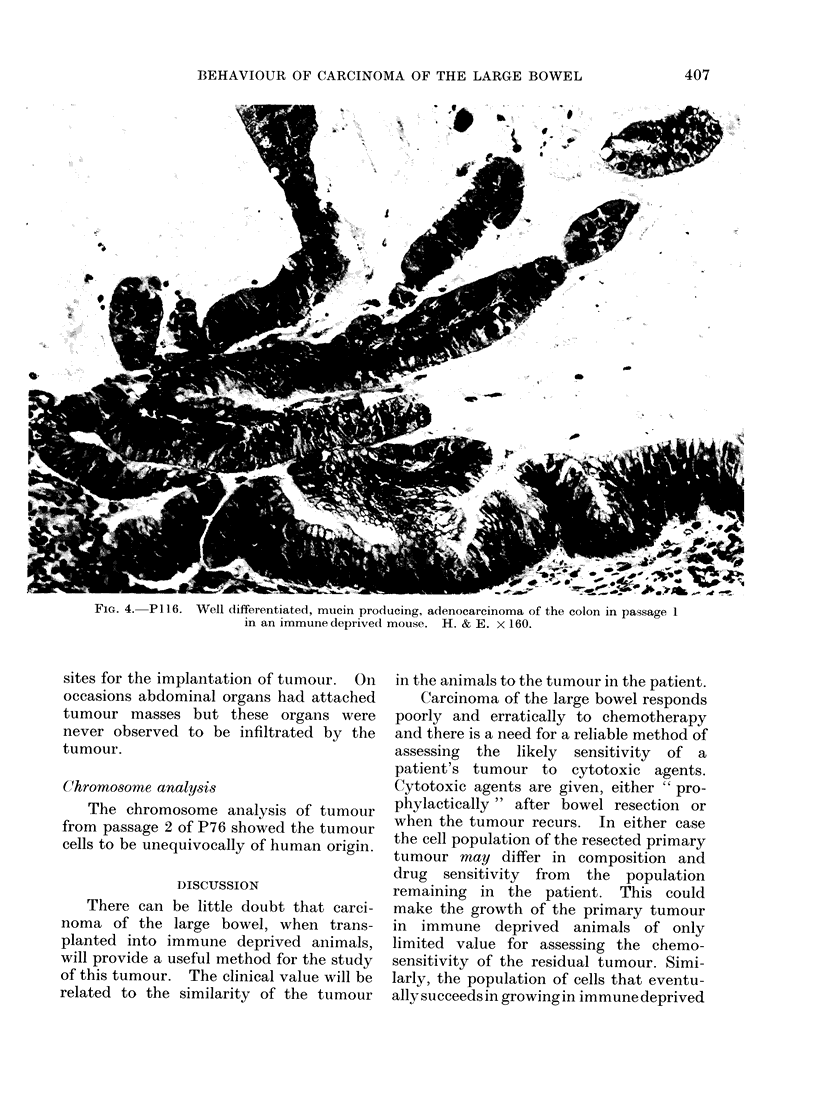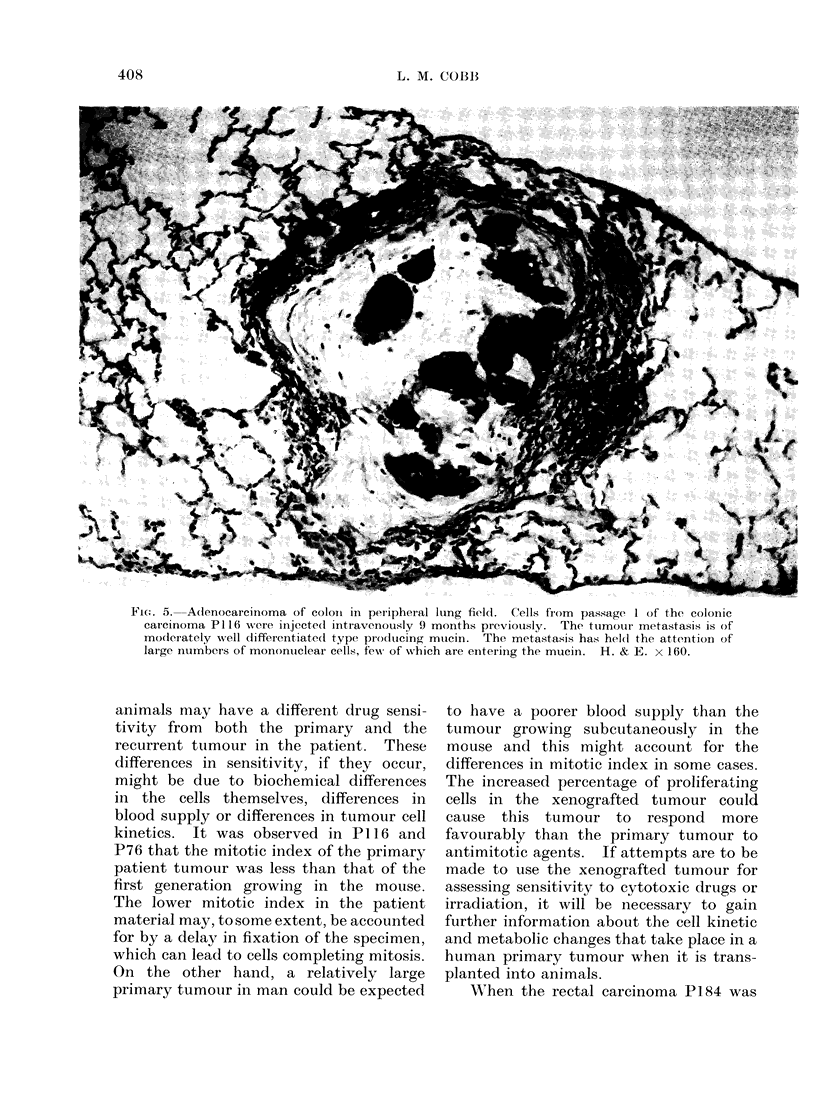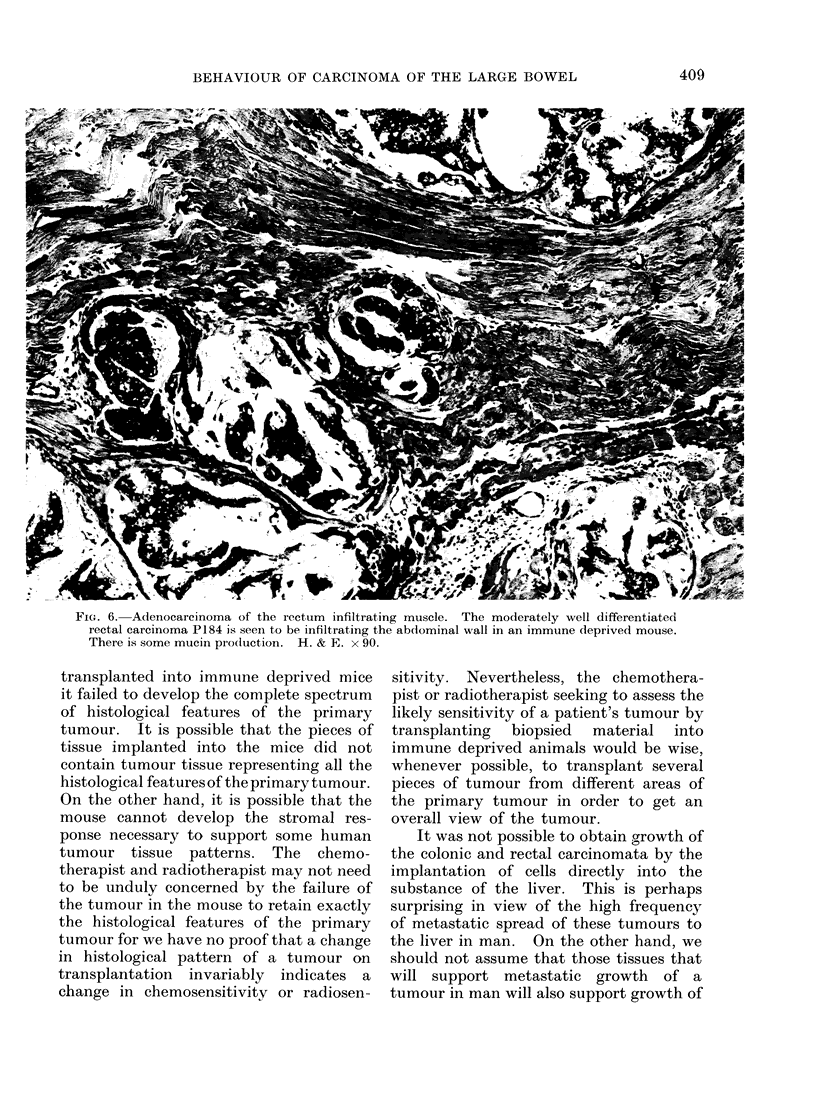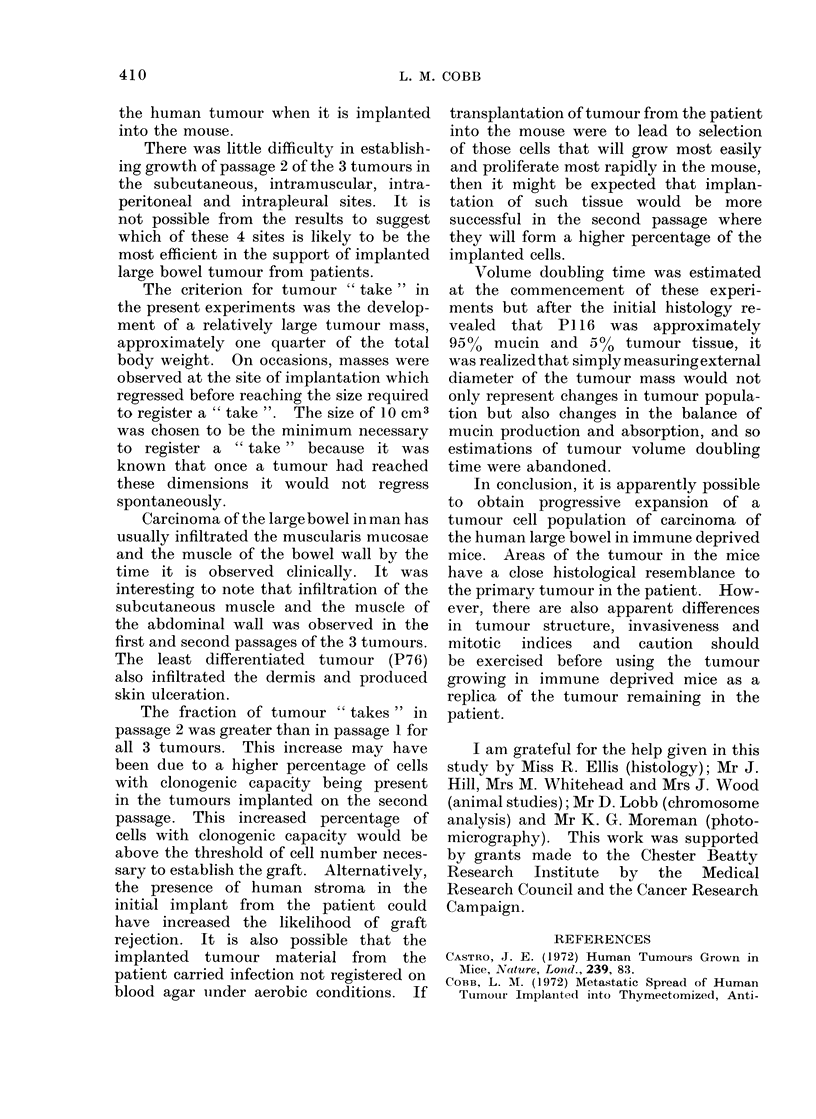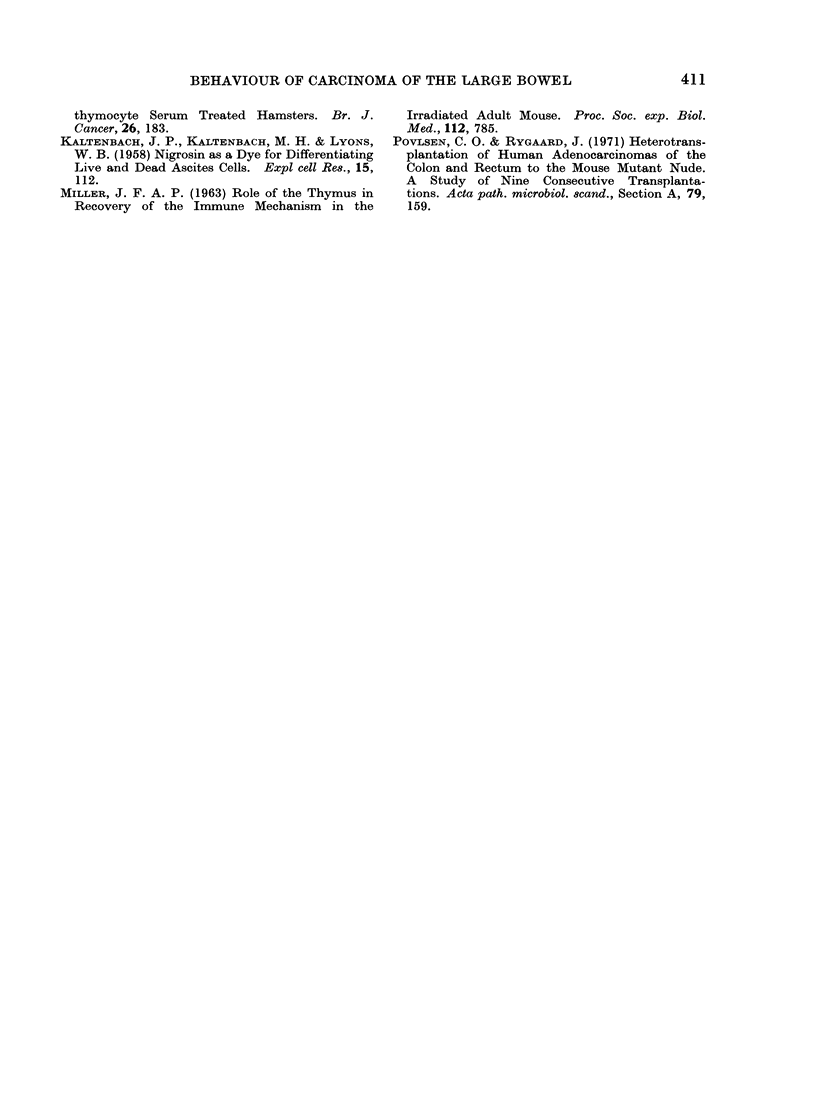# The Behaviour of Carcinoma of the Large Bowel in Man Following Transplantation into Immune Deprived Mice

**DOI:** 10.1038/bjc.1973.165

**Published:** 1973-11

**Authors:** L. M. Cobb

## Abstract

**Images:**


*Br. J. Cancer* (1973) **28**, 400

### THE BEHAVIOUR OF CARCINOMA OF THE LARGE BOWEL IN MAN FOLLOWING TRANSPLANTATION INTO IMMUNE DEPRIVED MICE

L. M. COBB*

*From the Chester Beatty Research Institute, Institute of Cancer Research, Royal Cancer Hospital, Fulham Road, London SW3 6JB*

Received 25 June 1973. Accepted 21 July 1973

**Summary.—The growth of carcinoma of the human large bowel was studied in the first 2 passages in immune deprived mice. The tumours were obtained from large bowel resections on 3 people. There was a strong histological similarity between the patient's tumour and the tumour that grew subcutaneously in the mice 2–8 months after implantation. One dissimilarity observed was a higher mitotic index in some of the tumours growing in the immune deprived animals. In the second passage of the bowel tumours, cells were implanted into groups of 8–10 animals in the following sites: subcutaneous, intramuscular, intravenous, intrahepatic, intraperitoneal and intrathoracic. Growth of tumour was observed from all 3 tumours when they were implanted subcutaneously, intramuscularly, intraperitoneally and intrathoracically. Infiltration of muscle by tumour was a frequent finding. Lung metastases developed after intravenous injection of cells in 1 of the 3 tumours. In none of the 3 tumours did growth follow injection of cells directly into the substance of the liver. On no occasions were spontaneous metastases observed.**

CARCINOMA arising in the large bowel in man can be transplanted successfully subcutaneously in immune deprived animals (Povlsen and Rygaard, 1971; Castro, 1972; Cobb, 1972). If we wish to use this xenograft model to learn more about a tumour in man, we need to understand to what extent transplantation to a xenogeneic host alters the tumour cell population. The work described in this article was designed to study the changes likely to occur when carcinoma of the large bowel is transplanted into immune deprived mice and, in addition, when this first passage tumour population is transplanted into a further group of immune deprived mice. The study of the second passage was instituted because occasions could be foreseen when insufficient of the primary tumour would be available to carry out all studies on the first passage.

In the present study passage 1 of the tumour was only carried subcutaneously, using a small amount of primary (patient's) tumour. When the tumour material had grown to a sufficient volume it was prepared for a second passage; on this occasion the tumour was implanted subcutaneously, intramuscularly, intravenously, intrahepatically and by injection into the pleural and peritoneal cavities. The results, in terms of tumour growth and invasiveness, are recorded below and the subsequent discussion is concerned with the problems of interpreting the behaviour of the tumour in mice in terms of its likely behaviour had those cells been allowed to remain in the human host.

### MATERIALS AND METHODS

*Experimental animals.*—Male and female CBA/lac mice were used. The breeding

* Present address: Huntingdon Research Centre, Huntingdon PE18 6ES.

stock was obtained from the Laboratory Animals Centre, Carshalton, Surrey, England. The mice were given sterile food and sterile water *ad libitum*. The sex of the animals was chosen to match the sex of the patient.

*Technique of immune deprivation.*—The technique used was based on that developed by Miller in this Institute and published in 1963, although in the present work younger animals and a higher dose of irradiation were used. The mice were thymectomized at 4 weeks of age and 4 weeks later they were given 900 rad whole body irradiation. Irradiation was given at 60 rad/min using a 220 kV x-ray machine, h.v.l. 0·4 $mm^3$, focal distance 100 cm. Without a bone marrow graft this dose of irradiation would have been lethal. Within 6 hours of irradiation each mouse received $5 \times 10^6$ syngeneic femoral bone marrow cells intravenously. The animals received the tumour graft between 2 and 4 weeks after irradiation.

*Source of tumours*

*Patient W.H.* (*Tumour number P*76).—The patient was a 66-year old married woman who had complained of tiredness and weakness for 8 months, and diarrhoea for 1 month. On admission to hospital she had a large pelvic mass and was very anaemic. At laparotomy a partial colectomy was performed for carcinoma of the colon. Because of involvement of other organs it was necessary to carry out partial ileectomy and partial cystectomy. The patient died 2 months later from bronchopneumonia and recurrent carcinoma of the colon. The tumour removed at operation was a poorly differentiated adenocarcinoma. There was infiltration of the wall of the large and small intestine and of the bladder. There was invasion by tumour of lymphatic vessels and veins. The regional lymph nodes were also involved with tumour.

*Patient R.G.* (*Tumour number P*116).—The patient was a 65-year old male who had complained of pain and swelling of the abdomen for 2 months. Diarrhoea had been intermittent but without blood. A left hemicolectomy was performed for adenocarcinoma of the colon. The tumour was a well differentiated, mucus producing, adenocarcinoma and had metastasized to the regional lymph node. Two years later (June, 1973) the patient is alive and well.

*Patient I.S.* (*Tumour number P*184).—The patient was a 72-year old spinster who had had intermittent diarrhoea for 6 months. Rectal bleeding had been observed for this period. Hartman's operation for resection of the rectum was performed for carcinoma of the rectum. The tumour was a poorly differentiated adenocarcinoma and had spread directly to involve the uterus. The patient recovered well from the operation but was lost to follow up.

*First* (*subcutaneous*) *tumour passage in animals*

Two of the 3 tumours (P76 and P184) were obtained from the operating theatre and implanted in the animals within 3 hours of resection. The third tumour (P116) was held at −190°C in 10% dimethyl sulphoxide and 90% Fischer's medium for 3 months before implantation. Slices of tumour each approximately $3 \times 3 \times 1$ mm were selected from different regions of each tumour and one slice of tumour implanted subcutaneously into the right flank of each of 5–8 mice (Table I). Adjacent pieces of tumour were fixed in Bouin's solution and prepared for histological examination.

In the first passage in all tumour bearing mice, when the tumour had reached approximately 10 $cm^3$ 95% of the tumour mass was removed together with an area of overlying skin. A sample of tumour, including skin, was taken for histological study. All animals were allowed to survive until the local recurrence of tumour necessitated their destruction. At necropsy the tumour and adjacent skin and muscle were retained for histological examination together with the axillary lymph nodes, lungs (inflated with fixative), heart, thyroid, liver, suparenals, kidneys, testes or ovaries, pancreas, small and large intestine. The tissues were fixed in Bouin's fluid.

*Preparation of tumour cell suspension for injection.*—Enzymes were used to separate passage 1 tumour cells for injection into a second group of mice to give passage 2. Finely chopped tumour was incubated for 2 hours in a solution of TC199 containing 0·25% ovine hyaluronidase (Seravac Laboratories, Maidenhead, Berkshire, England), and 0·25% collagenase (Sigma Chemical Company, St Louis, U.S.A.). After 2 hours incubation the tissue preparation was passed through a gauze filter to remove the larger pieces of stroma and tumour and the resulting suspension of cells was washed 3 times in phosphate buffered saline before injection into mice.

TABLE I.—*Growth of Carcinoma of Colon and Rectum Implanted Subcutaneously into Immune Deprived Mice*

| Tumour No. | Histology | " Takes "* | Time (months) from 9 mm³ to 10 cm³ —passage 1† | Mitotic index‡ Patient tumour | Passage 1 (S/C)§ | Passage 2 (S/C) |
|---|---|---|---|---|---|---|
| | | | | % | % | % |
| P76 | Poorly differentiated adenocarcinoma of colon | 2/8 | 3–8 | 0·5 | 3·2 | 3 |
| P116 | Well differentiated adenocarcinoma of colon | 3/5 | 5–8 | 0·1 | 0·4 | 0·4 |
| P184 | Moderately well differentiated adenocarcinoma of rectum | 3/8 | 6–7 | 0·4 | 0·3 | 0·4 |

* A "take " was recorded if the tumour grew to 10 $cm^3$.
† Passage 1 was the growth of tumour implanted from the patient.
‡ The mitotic indices were of the first tumour to grow to 10 $cm^3$ in passages 1 and 2. $1 \times 10^4$ cells were counted to estimate a mitotic index.
§ S/C subcutaneous tumour.

TABLE II.—*Number of " Takes " in Passage 2 of P76, P116 and P184 for 6 Sites of Implantation*

| Site and nature of implant | Takes* (passage 2) P76 | P116 | P184 |
|---|---|---|---|
| Subcutaneous ($3 \times 3 \times 1$ mm) | 8/10 | 10/10 | 6/10 |
| Intramuscular ($3 \times 1 \times 1$ mm) | 10/10 | 10/10 | 10/10 |
| Intraperitoneal ($1 \times 10^6$ viable cells) | 8/10 | 10/10 | 9/9 |
| Intrapleural ($1 \times 10^6$ viab[illegible] [illegible]lls) | 8/8 | 9/9 | 6/9 |
| Intrahepatic ($2 \times 10^5$ viable cells) | 0/10 | 0/10 | 0/10 |
| Intravenous ($2 \times 10^5$ viable cells) | 0/10 | 4/10 | 0/10 |

* A " take " was recorded when the extent of tumour growth necessitated the destruction of the animal, except in the case of intravenous injection of cells when " takes " were recorded when there was macroscopic and/or microscopic evidence of one or more lung metastases at the termination of the experiment.

The viability of cells was assessed microscopically by their ability to resist staining with nigrosin (Kaltenbach, Kaltenbach and Lyons, 1958).

*Second tumour passage in animals*

The first mouse to bear a tumour of 10 $cm^3$ in passage 1 of each of the 3 implanted tumours (P76, P116, P184) supplied all the tumour tissue for the study of the second passage of tumour (passage 2). Either pieces of tumour, or a cell suspension of tumour, from these 3 mice were implanted into groups of 8, 9, or 10 immune deprived mice by one of the following routes: subcutaneous, intramuscular, intrahepatic, intravenous, intraperitoneal or intrapleural (intrathoracic) (Table II). No animal was implanted with tumour in more than one site.

For the subcutaneous implantation slices of tumour, $3 \times 3 \times 1$ mm, were implanted into the right flank. Pieces of tumour, $3 \times 1 \times 1$ mm, were implanted intramuscularly in the quadriceps group of the right hind leg of other groups of mice. Cell suspensions containing single cells and clumps of up to 10 cells were inoculated intraperitoneally ($1 \times 10^6$ viable cells), intrapleurally ($1 \times 10^6$ viable cells), intravenously ($2 \times 10^5$ viable cells) and directly into the substance of the liver during laparotomy ($2 \times 10^5$ viable cells). Post mortem examinations were carried out on all animals, when they became moribund, or 9 months after implantation when all the remaining animals were killed at the termination of the experiment. All organs were examined for macroscopic pathology and the following organs taken for microscopic examination: lung, heart, thyroid, ovary or testis, suprarenal, kidney, liver, pancreas, small and large intestine. The tissues were fixed in Bouin's fluid. Sections were cut, and stained with haematoxylin and eosin and/or periodic acid–Schiff. The numbers of animals inoculated and the numbers of animals subsequently bearing tumours are given in Table II.

*Chromosome analysis.*—A mouse bearing subcutaneously the tumour P76 in passage 2 was injected subcutaneously with 0·1 mg of Colcemid (Ciba Pharmaceutical Products, Basle, Switzerland). Two hours later the tumours was dissected from the animal and a tumour cell separate made by mechanically forcing the tumour through a fine metal sieve. A preparation of these tumour cells was made for chromosome analysis in order to confirm that the tumour was of human origin.

*Bacteriological study.*—Tissue adjacent to those areas of tumour selected for transplantation in all experiments were smeared onto blood agar plates and incubated aerobically for 72 hours.

### RESULTS

### *Behaviour of P76 in immune deprived mice*

The tumour grew progressively in 2 of the 8 immune deprived mice into which pieces of the primary tumour were implanted (Table I). After 3 months the most rapidly growing tumour had reached an estimated volume of 10 $cm^3$ and this tumour material was used for the passage 2 studies. The tumour in the second mouse did not reach 10 $cm^3$ until 8 months after implantation. The histological appearance of tumour removed from the 2 mice closely resembled that of the patient's tumour. Many areas of the tumour in the mice were indistinguishable histologically from the primary tumour in the patient. The mitotic index of the tumour material from the mouse was noticeably higher than the mitotic index in the patient's tumour (Table I). The tumour growing subcutaneously in the mice had infiltrated and destroyed areas of the subcutaneous muscle and in one animal this led to ulceration of the skin (Fig. 1). There was no evidence of infiltration of lymphatic vessels or blood vessels by the tumour in the mouse, although this had been the case in the primary tumour when growing in the patient.

In the second generation of subcutaneous passage in mice (passage 2) 8 of the 10 implanted mice grew tumours (Table II). The first tumour to grow to 10 $cm^3$ did so 2 months after the implantation of a 9 $mm^3$ piece of tumour. The histological appearance was similar to the tumour of passage 1 with a similar mitotic index (Table I). There was infiltration by the tumour of the underlying abdominal muscle (Fig. 2). In none of the subcutaneously implanted mice from passages 1 and 2 were regional lymph nodes involved with tumour cells.

The attempts to establish growth of the tumour P76 in mice by intravenous injection of cells, or injection of cells directly into the liver, were without success. Following injection of tumour cells directly into the pleural cavity, the tumour grew extensively on the parietal pleura and only occasionally on the visceral pleura. The histological appearance of the tumour was essentially that of the patient's tumour, although the stromal response appeared to be less than that of the patient's tumour. There appeared to be some selection of cells capable of forming isolated hollow spheres of tumour cells, having only a small area of attachment to the pleura. Following intraperitoneal injection of the cell suspension, the tumour became established mostly on the greater and lesser omenta and the peritoneal surface of the diaphragm. Occasionally other areas of the parietal and visceral peritoneum provided sites for attachment of tumour. As with the growth in the thorax, the tumour was supported by a minimum of stroma. There was no histological evidence of infiltration of the abdominal organs but the track of the injecting needle through the abdominal wall was frequently identified by infiltration of abdominal muscle

FIG. 1.—The infiltration of subcutaneous muscle and extension of tumour (P76) into the dermis at the periphery of an area of ulceration of the skin in the mouse. Polymorph infiltration at the site of ulceration indicates tissue necrosis and perhaps infection. The dermis encapsulating the tumour is infiltrated with mononuclear cells and a small number of polymorphs. H. & E. ×196.

by tumour. The tumour implanted into the muscle of the hind limb grew in all animals, illustrating clearly the ability of the tumour to grow and to infiltrate striated muscle in the mouse. Tumour was not observed in the regional lymph nodes of the hind limbs.

*Behaviour of P116 in immune deprived mice*

The tumour implanted into 5 immune deprived mice grew progressively in 3 of the animals and grew for only a short period, before regressing, in the other 2 animals (Table I). The growth of tumour and subsequent regression in these 2 animals was assessed only by palpation; a biopsy was not taken to confirm the presence of tumour. In the 3 mice in which the tumour grew progressively the histology was that of a well differentiated, mucus secreting adenocarcinoma of the colon. Its appearance was very similar to the primary tumour in the patient (Fig. 3 and 4). The tumour growing in the mice appeared to have greater lymphocyte infiltration than did the patient's tumour although the difference was marginal. As with P76, the mitotic index of passage 1 of P116 in the mice was significantly higher than in the patient (Table I). Passage 2 of P116 in the mice grew in 10 out of 10 subcutaneously implanted animals (Table II). The tumour reached a volume of 10 cm$^3$ in some of the 10 mice more quickly in passage 2 than in passage

FIG. 2.—Infiltration of the muscle of the abdominal wall of the mouse by carcinoma of the colon (P76). The tumour has infiltrated between individual muscle fibres of the abdominal wall. H. & E. × 235.

1. However, the extensive formation of mucin and the resultant uncertainty of the exact cellular content of the implanted tumour pieces made measurement of tumour volume and estimation of tumour volume doubling time of little value. The subcutaneous and intramuscular growth of passage 2 showed the ability of this tumour to infiltrate adjacent muscle.

The tumour grew well when injected as a cell suspension intrapleurally and intraperitoneally. Attachment was predominantly to the parietal pleura and diaphragm in the thorax and to the visceral peritoneum, particularly the omentum in the abdomen. It was not possible to obtain growth of the tumour by injection of $2 \times 10^5$ live cells into the substance of the liver. In 4 of the 10 mice receiving $2 \times 10^5$ cells intravenously, scattered lung metastases were observed macroscopically when the 10 mice were killed 9 months after the intravenous injection of the cells. The metastases appeared microscopically as small groups of well differentiated adenocarcinoma cells suspended in mucin (Fig. 5).

*Behaviour of P184 in immune deprived mice*

The rectal tumour P184 grew progressively in 3 of 8 immune deprived mice (Table I). The first tumour reached 10 $cm^3$ 6 months after implantation. This tumour material was used for passage 2. The passage 1 tumour in all 3 mice grew as a moderately well differentiated rectal carcinoma. In parts of the passage

FIG. 3.—P116. An area of well differentiated, mucin producing, adenocarcinoma in the patient R.G. H. & E. ×160.

1 tumour, groups of signet ring cells were observed floating in pools of mucin. The pools of mucin were separated by thin connective tissue septa. In other areas the mucin secretion was negligible. This histological appearance was also present in sections from the primary tumour in the patient. In addition, the patient's tumour contained areas of well differentiated rectal carcinoma showing very little loss of nuclear polarity and extensive stromal support. Such areas were not represented in the tumour growing in the immune deprived mice. The mitotic index of the subcutaneous passages 1 and 2 of P184 in mice was not significantly different from that of the primary tumour. There was infiltration of subcutaneous and abdominal musculature by the tumour on passage 1 (Fig. 6) and passage 2 but no evidence of infiltration of lymphatic vessels or veins and no metastatic spread to regional lymph nodes.

Efforts to establish growth of P184 in immune deprived mice by intrahepatic and intravenous injection of cells failed (Table II). As with P76 and P116, the tumour grew successfully following intrapleural and intraperitoneal inoculation. Intrapleurally, the tumour grew on the parietal pleura which provided stromal support for growing clumps of moderately well differentiated rectal carcinoma cells. In the mice killed because of extensive intraperitoneal growth the visceral peritoneum and diaphragm provided major

FIG. 4.—P116. Well differentiated, mucin producing, adenocarcinoma of the colon in passage 1 in an immune deprived mouse. H. & E. ×160.

sites for the implantation of tumour. On occasions abdominal organs had attached tumour masses but these organs were never observed to be infiltrated by the tumour.

*Chromosome analysis*

The chromosome analysis of tumour from passage 2 of P76 showed the tumour cells to be unequivocally of human origin.

### DISCUSSION

There can be little doubt that carcinoma of the large bowel, when transplanted into immune deprived animals, will provide a useful method for the study of this tumour. The clinical value will be related to the similarity of the tumour in the animals to the tumour in the patient.

Carcinoma of the large bowel responds poorly and erratically to chemotherapy and there is a need for a reliable method of assessing the likely sensitivity of a patient's tumour to cytotoxic agents. Cytotoxic agents are given, either " prophylactically " after bowel resection or when the tumour recurs. In either case the cell population of the resected primary tumour *may* differ in composition and drug sensitivity from the population remaining in the patient. This could make the growth of the primary tumour in immune deprived animals of only limited value for assessing the chemosensitivity of the residual tumour. Similarly, the population of cells that eventually succeeds in growing in immune deprived

FIG. 5.—Adenocarcinoma of colon in peripheral lung field. Cells from passage 1 of the colonic carcinoma P116 were injected intravenously 9 months previously. The tumour metastasis is of moderately well differentiated type producing mucin. The metastasis has held the attention of large numbers of mononuclear cells, few of which are entering the mucin. H. & E. × 160.

animals may have a different drug sensitivity from both the primary and the recurrent tumour in the patient. These differences in sensitivity, if they occur, might be due to biochemical differences in the cells themselves, differences in blood supply or differences in tumour cell kinetics. It was observed in P116 and P76 that the mitotic index of the primary patient tumour was less than that of the first generation growing in the mouse. The lower mitotic index in the patient material may, to some extent, be accounted for by a delay in fixation of the specimen, which can lead to cells completing mitosis. On the other hand, a relatively large primary tumour in man could be expected to have a poorer blood supply than the tumour growing subcutaneously in the mouse and this might account for the differences in mitotic index in some cases. The increased percentage of proliferating cells in the xenografted tumour could cause this tumour to respond more favourably than the primary tumour to antimitotic agents. If attempts are to be made to use the xenografted tumour for assessing sensitivity to cytotoxic drugs or irradiation, it will be necessary to gain further information about the cell kinetic and metabolic changes that take place in a human primary tumour when it is transplanted into animals.

When the rectal carcinoma P184 was

FIG. 6.—Adenocarcinoma of the rectum infiltrating muscle. The moderately well differentiated rectal carcinoma P184 is seen to be infiltrating the abdominal wall in an immune deprived mouse. There is some mucin production. H. & E. × 90.

transplanted into immune deprived mice it failed to develop the complete spectrum of histological features of the primary tumour. It is possible that the pieces of tissue implanted into the mice did not contain tumour tissue representing all the histological features of the primary tumour. On the other hand, it is possible that the mouse cannot develop the stromal response necessary to support some human tumour tissue patterns. The chemotherapist and radiotherapist may not need to be unduly concerned by the failure of the tumour in the mouse to retain exactly the histological features of the primary tumour for we have no proof that a change in histological pattern of a tumour on transplantation invariably indicates a change in chemosensitivity or radiosensitivity. Nevertheless, the chemotherapist or radiotherapist seeking to assess the likely sensitivity of a patient's tumour by transplanting biopsied material into immune deprived animals would be wise, whenever possible, to transplant several pieces of tumour from different areas of the primary tumour in order to get an overall view of the tumour.

It was not possible to obtain growth of the colonic and rectal carcinomata by the implantation of cells directly into the substance of the liver. This is perhaps surprising in view of the high frequency of metastatic spread of these tumours to the liver in man. On the other hand, we should not assume that those tissues that will support metastatic growth of a tumour in man will also support growth of the human tumour when it is implanted into the mouse.

There was little difficulty in establishing growth of passage 2 of the 3 tumours in the subcutaneous, intramuscular, intraperitoneal and intrapleural sites. It is not possible from the results to suggest which of these 4 sites is likely to be the most efficient in the support of implanted large bowel tumour from patients.

The criterion for tumour " take " in the present experiments was the development of a relatively large tumour mass, approximately one quarter of the total body weight. On occasions, masses were observed at the site of implantation which regressed before reaching the size required to register a " take ". The size of 10 $cm^3$ was chosen to be the minimum necessary to register a " take " because it was known that once a tumour had reached these dimensions it would not regress spontaneously.

Carcinoma of the large bowel in man has usually infiltrated the muscularis mucosae and the muscle of the bowel wall by the time it is observed clinically. It was interesting to note that infiltration of the subcutaneous muscle and the muscle of the abdominal wall was observed in the first and second passages of the 3 tumours. The least differentiated tumour (P76) also infiltrated the dermis and produced skin ulceration.

The fraction of tumour " takes " in passage 2 was greater than in passage 1 for all 3 tumours. This increase may have been due to a higher percentage of cells with clonogenic capacity being present in the tumours implanted on the second passage. This increased percentage of cells with clonogenic capacity would be above the threshold of cell number necessary to establish the graft. Alternatively, the presence of human stroma in the initial implant from the patient could have increased the likelihood of graft rejection. It is also possible that the implanted tumour material from the patient carried infection not registered on blood agar under aerobic conditions. If transplantation of tumour from the patient into the mouse were to lead to selection of those cells that will grow most easily and proliferate most rapidly in the mouse, then it might be expected that implantation of such tissue would be more successful in the second passage where they will form a higher percentage of the implanted cells.

Volume doubling time was estimated at the commencement of these experiments but after the initial histology revealed that P116 was approximately 95% mucin and 5% tumour tissue, it was realized that simply measuring external diameter of the tumour mass would not only represent changes in tumour population but also changes in the balance of mucin production and absorption, and so estimations of tumour volume doubling time were abandoned.

In conclusion, it is apparently possible to obtain progressive expansion of a tumour cell population of carcinoma of the human large bowel in immune deprived mice. Areas of the tumour in the mice have a close histological resemblance to the primary tumour in the patient. However, there are also apparent differences in tumour structure, invasiveness and mitotic indices and caution should be exercised before using the tumour growing in immune deprived mice as a replica of the tumour remaining in the patient.

I am grateful for the help given in this study by Miss R. Ellis (histology); Mr J. Hill, Mrs M. Whitehead and Mrs J. Wood (animal studies); Mr D. Lobb (chromosome analysis) and Mr K. G. Moreman (photomicrography). This work was supported by grants made to the Chester Beatty Research Institute by the Medical Research Council and the Cancer Research Campaign.

REFERENCES

Castro, J. E. (1972) Human Tumours Grown in Mice, *Nature, Lond.*, **239**, 83.

Cobb, L. M. (1972) Metastatic Spread of Human Tumour Implanted into Thymectomized, Antithymocyte Serum Treated Hamsters. *Br. J. Cancer*, **26**, 183.

KALTENBACH, J. P., KALTENBACH, M. H. & LYONS, W. B. (1958) Nigrosin as a Dye for Differentiating Live and Dead Ascites Cells. *Expl cell Res.*, **15**, 112.

MILLER, J. F. A. P. (1963) Role of the Thymus in Recovery of the Immune Mechanism in the Irradiated Adult Mouse. *Proc. Soc. exp. Biol. Med.*, **112**, 785.

POVLSEN, C. O. & RYGAARD, J. (1971) Heterotransplantation of Human Adenocarcinomas of the Colon and Rectum to the Mouse Mutant Nude. A Study of Nine Consecutive Transplantations. *Acta path. microbiol. scand.*, Section A, **79**, 159.